# Effect of ligands and HIV latency-reversing agents on estrogen receptor **α** in CD4^+^ T cells

**DOI:** 10.1172/jci.insight.198415

**Published:** 2026-07-02

**Authors:** Cristina Ceriani, Priya Khetan, Anthony Abeyta-Lopez, Kena J. Lemu, Prachi Meher, Brigitte Allard, Katherine S. James, Anne-Marie W. Turner, David M. Margolis, Nancie M. Archin

**Affiliations:** 1 UNC HIV Cure Center, and; 2 Department of Medicine, The University of North Carolina (UNC) at Chapel Hill, Chapel Hill, North Carolina, USA.; 3 Department of Microbiology and Immunology, UNC at Chapel Hill, Chapel Hill, North Carolina, USA.; 4 Division of Translational Informatics, Department of Internal Medicine, University of New Mexico, Albuquerque, New Mexico, USA.

**Keywords:** AIDS/HIV, Cell biology, Infectious disease, Sex hormones

## Abstract

The estrogen receptor (ER) is hypothesized to directly influence HIV transcription and latency but is also critical for immune signaling. However, the mechanisms of action of the ER in immune cells in the context of HIV are limited, and relevant to HIV cure strategies, the influence of latency reversal agents (LRAs) on the ER pathway are unknown. We evaluated (a) the effect of estrogen (E2) on the nuclear translocation of ERα in CD4^+^ T cells; (b) the ability of Fulvestrant, a selective estrogen receptor degrader (SERD), and ARV-471, a potent, PROteolysis TArgeting Chimera (PROTAC) selective ERα degrader to modulate ERα; and c) the effect of different classes of LRAs on ERα signaling. In contrast to what has been demonstrated in oncology, E2 did not induce ERα nuclear translocation in CD4^+^ T cells. Similarly, neither Fulvestrant nor ARV-471 induced degradation of ERα in CD4^+^ T cells. LRAs significantly downregulated ERα gene and protein expression in both PBMCs and CD4^+^ T cells. Collectively, our results suggest that estrogen influences on HIV transcription are not likely a consequence of canonical nuclear ERα mechanisms. The consequences of LRA downregulation of ERα, a protein important for immune signaling, warrant further investigation.

## Introduction

Effective HIV prevention approaches along with increased access to diagnosis, treatment, and care have led to continued decline in the incidence of new HIV infection across the globe (1). Since 2010, new HIV infections have declined by 38%. However, despite these remarkable advances, HIV remains a considerable global public health concern and threat. Currently, approximately 39 million people are living with HIV globally and while until recently 76% of people living with HIV (PLWH) had access to antiretroviral therapy (ART), which with proper adherence can suppress HIV replication and reduce HIV transmission (1), ART is not curative. Cellular reservoirs of latent, replication-competent HIV persist despite successful ART and are capable of reigniting uncontrolled infection. These reservoirs of viruses constitute a major challenge toward achieving an HIV cure and require lifelong access to therapy for millions of people for decades to come. Thus, a cure is desperately needed, as HIV disease is a life-long infection and a substantial number of PLWH lack access to ART. Importantly, over 50% of PLWH infection globally are women who are traditionally underenrolled in cure research (2). Knowledge of sex-specific factors influencing HIV persistence could foster the development of efficacious strategies to deplete the reservoir in all people.

Hormonal and genetic factors can affect the immune response to pathogens, including HIV, which can in turn affect HIV persistence and control in women differently than in men. Sex differences in type I IFN response, for instance, can limit HIV infection initially but can exacerbate infection with an increased in chronic immune activation in women (3, 4). More recently, several reports argue for sex-based differences in reservoir size in some but not all cohorts of PLWH, with a lower inducible reservoir reported in women (5–7). Hormonal influence on HIV transcription has also been reported, with estrogen thought to induce a repressive environment for HIV transcription (8, 9). This is important as modulation of epigenetic or signaling pathways involved in HIV transcriptional suppression using latency reversal agents (LRAs) to reactivate latent HIV has emerged as an important HIV cure strategy; thus, estrogen could negatively affect the efficacy of LRAs.

Estrogen signaling plays important roles in immune modulation in a cell type–dependent manner which could also affect the clearance of cells containing reactivated virus (10). For example, NK cell activity and proliferation is reported to be influenced by estrogen during pregnancy with decreased activity reported during the third trimester when estrogen levels are high (11). Furthermore, treatment of mice with exogenous estrogen leads to decreased expression of activation markers on NK cells with concomitant decrease in cytotoxicity (12). The effects of estrogen on T cells are extensive. Estrogen can affect T cell development and maturation in the thymus as well as influence Treg differentiation and function (13, 14). Estrogen is reported to affect CD8^+^ T cell activity through its effect on CD4^+^ T cell differentiation in a dose-dependent manner with low levels of estrogen favoring a Th1 response and IFN-γ production and higher levels favoring a Th2 response (reviewed in ref. 10). Furthermore, estrogen can also affect the secretion of other cytokines in CD4^+^ T cells as well as influence the responsiveness of these cells to TCR stimulation (15, 16). Estrogen mediates signaling predominantly through the estrogen receptors α and β (ERα and ERβ, respectively), which are intracellular. ERα and ERβ are members of the nuclear hormone receptor superfamily and are encoded by the *ESR1* and *ESR2* genes, respectively. Although both receptors are expressed on immune cells, among lymphocytes, CD4^+^ T cells express higher levels of *ESR1* mRNA than *ESR2* (17, 18). Recent studies have identified the G-protein coupled estrogen receptor 1 (GPER1, formerly GPR30) as a transmembrane estrogen receptor involved in rapid estrogen response (19). However, GPER1 has a relatively low affinity for estrogen, and as mentioned above, it is believed that estrogen signaling occurs mainly through ERα and ERβ.

Given the importance of estrogen signaling on immunity and HIV transcription, and with latency reversal approaches to deplete the HIV reservoir being widely tested in clinical settings, a clearer understanding of the interplay of estrogen receptor signaling and latency reversal may help guide future therapies seeking to eliminate persistent HIV infection, particularly as these strategies are applied to women. In this study, we sought to define the effect of LRAs on ERα signaling in CD4^+^ T cells obtained from seronegative individuals and PLWH durably suppressed on ART. We observe that different classes of LRAs downmodulate the expression of ERα at both the mRNA and protein levels. Furthermore, we observed that, unlike in the T-47D breast cancer cell line, exposure of CD4^+^ T cells to exogenous estrogen did not lead to nuclear translocation of the receptor as expected. Similarly, in CD4^+^ T cells, ER degraders, such as ARV-471 — a potent, first-in-human proteolysis targeting chimera (PROTAC) — did not lead to a decrease in ERα protein as previously observed in breast cancer cells (20, 21). Our results suggest that LRAs directly affect ERα expression, which could in turn influence ERα-dependent gene transcription (e.g., pro- or antiinflammatory cytokines, or HIV), and this should be taken into consideration in HIV cure studies. Additionally, ERα signaling in CD4^+^ T cells in response to estrogen leading to HIV transcriptional suppression may occur through noncanonical ER signaling pathways.

## Results

### No biological sex differences observed in ESR1 gene expression.

**Figure 1:**
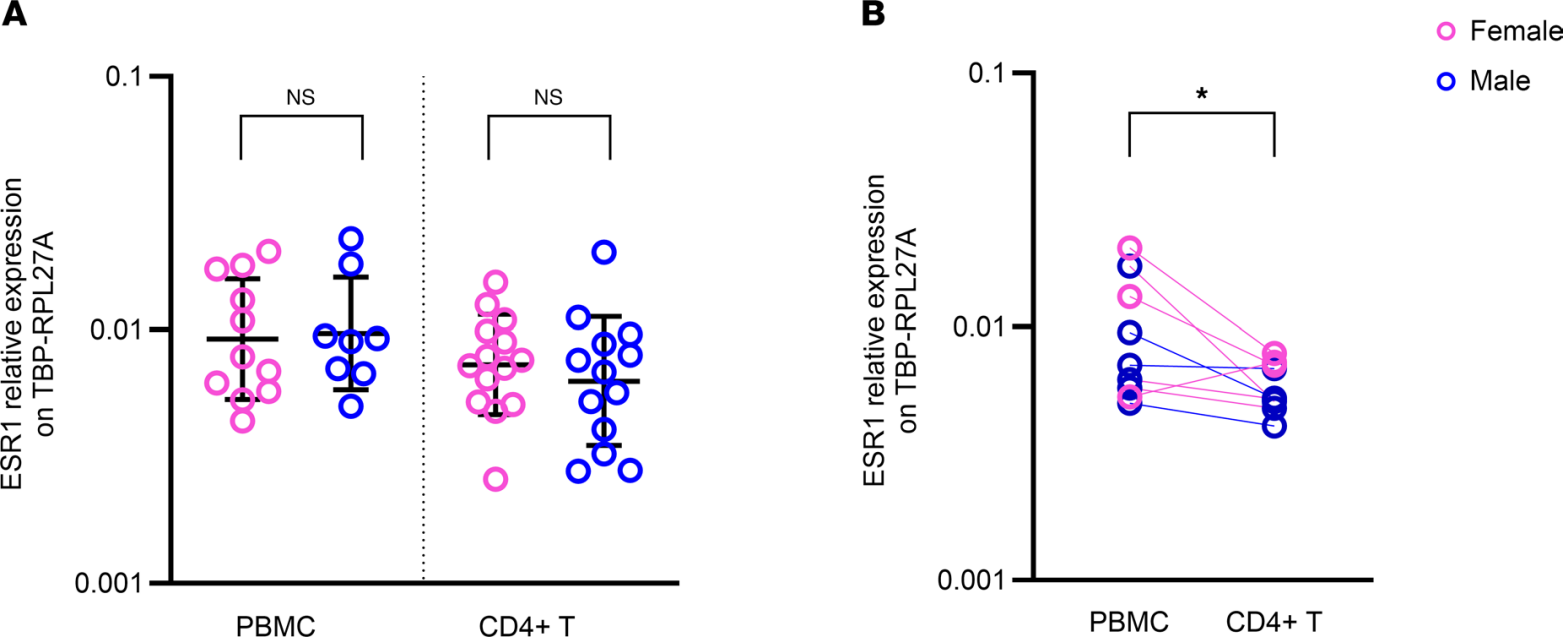
*ESR1* mRNA expression in male and female. (**A**) *ESR1* mRNA baseline expression in peripheral blood mononuclear cells (PBMCs) and purified CD4^+^ T cells from seronegative female (PBMC, *n* = 11; CD4, *n* = 14) and male donors (PBMC, *n* = 8; CD4, *n* = 13). (**B**) Paired *ESR1* mRNA expression in PBMCs and CD4^+^ T cells isolated from the same donors (*n* = 7). Each point represents an individual sample. Geometric mean with geometric SD shown. Statistical significance was calculated using Mann-Whitney *U* test or Wilcoxon test compared with DMSO as appropriate. *P < 0.05; **P < 0.005, ***P < 0.001.

As mentioned previously, while estrogen signals predominantly through ERα and ERβ, ERα is the predominant form expressed in CD4^+^ T cells and has been reported to have a suppressive effect on HIV transcription (8, 9). We therefore first evaluated gene expression levels of ERα and assessed any sex differences. We compared the gene expression of *ESR1* in peripheral blood mononuclear cells (PBMC) and CD4^+^ T cells from seronegative donors. Consistent with other studies in HIV seronegative individuals and PLWH, levels of *ESR1* mRNA expression in CD4^+^ T cells and in unfractionated total PBMC isolated from men and women were similar (Figure 1A) (5, 18). When comparing levels of expression between PBMC and isolated CD4^+^ T cells, we observed a modest but statistically significant increase in expression of *ESR1* mRNA in PBMC compared with CD4^+^ T cells (Figure 1B). This most likely reflects expression in cell types other than CD4^+^ T cells in unfractionated PBMC. ESR2 mRNA (encoding ERβ) levels were confirmed in unfractionated PBMC and CD4^+^ T cells and were comparatively lower than *ESR1* levels (not shown) as previously reported (17, 18).

### Downregulation of ESR1 gene expression by LRAs in PBMC and CD4^+^ T cells.

Given the extensive effect of estrogen signaling on immune function, and the widespread use of LRAs in clinical testing as part of HIV cure strategies, we next sought to determine whether LRA exposure causes any modulation of ERα. We evaluated the effect of LRAs on *ESR1* gene expression and ERα protein levels, in both, unfractionated PBMCs, and CD4+ T cells. LRAs evaluated included the T cell receptor activator phytohemagglutinin A (PHA), the PKC agonists PEP005 and phorbol-12-myristate-13-acetate with the calcium ionophore, ionomycin (PMAi), the HDAC inhibitors romidepsin (ROM) and vorinostat (VOR), the bromodomain inhibitor iBET-151 (iBET), the IAP inhibitor AZD-5582 (AZD), and a STING pathway agonist (22).

**Figure 2:**
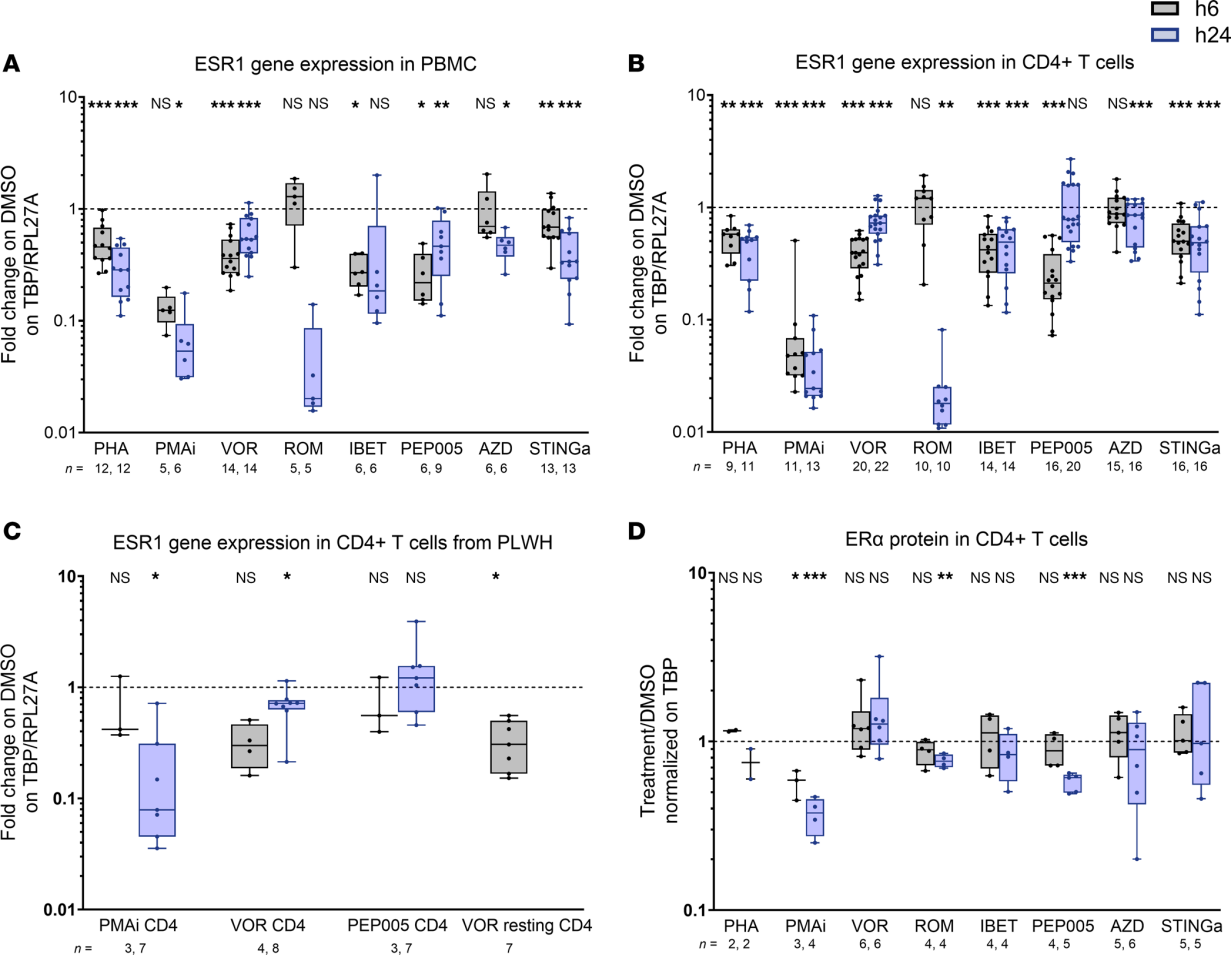
LRAs significantly downregulate ERα gene expression and decrease ERα protein. (**A**–**C**) *ESR1* mRNA fold expression, relative to DMSO, after 6 hours and 24 hours exposure with LRAs in PBMC (**A**) and CD4^+^ T cells (**B**) from seronegative individuals, and CD4^+^ T cells and resting CD4^+^ T cells from PLWH (**C**). (**D**) ERα protein quantification, relative to DMSO, after 6 hours and 24 hours exposure with LRAs in CD4^+^ T cells from seronegative individuals. Sample sizes (*n*) are indicated below each group on the *x* axis. Each point represents an individual sample. Boxes show the interquartile range with median; whiskers span the full range. Fold change is relative to DMSO, with the horizontal line at 1 indicating baseline; values > 1 reflect upregulation, < 1 reflect downregulation. Wilcoxon test was performed by comparing each LRA treatment to the untreated control (DMSO) at the same time point. *P < 0.05; **P < 0.005, ***P < 0.001.

To evaluate the effect of these commonly used LRAs on *ESR1* gene expression, we treated PBMC and CD4^+^ T cells with LRAs for 6 hours and 24 hours. *ESR1* mRNA was significantly downregulated by the majority of the LRAs tested in both PBMCs (Figure 2A) and CD4^+^ T cells (Figure 2B) compared with untreated conditions, with similar trends observed between the 2 cell populations. Given that the HIV reservoir is primarily contained within CD4 T cells, henceforth, we focused primarily on the CD4^+^ T cell population.

As shown in Figure 2B, at 6 hours, the highest *ESR1* mRNA downregulation was observed with PMAi (0.05-fold change, *P* < 0.001), while at 24-hours, ROM exhibited the highest downregulation (0.02-fold change, *P* < 0.01). Significant rapid downregulation was observed at 6-hour time point for all LRAs, except AZD treatment which results in minimal downregulation only at the 24-hour time point (0.73-fold change, *P* < 0.001) consistent with the slower onset of pathway signaling induced by AZD (23). The rapid downregulation observed at the 6-hour time point was followed by an incremental decrease at 24 hours for PHA, PMAi, and STINGa and a partial restoration for VOR and PEP005.

No significant sex differences were observed in *ESR1* downregulation in either CD4^+^ T cells or unfractionated PBMC (Supplemental Figure 1; supplemental material available online with this article; https://doi.org/10.1172/jci.insight.198415DS1).

These findings were recapitulated in HIV ART-suppressed donors, as shown in Figure 2C, specifically with VOR treatment for 6 hours in resting CD4^+^ T cells (rCD4) and VOR, PMAi, and PEP005 treatments for 24 hours in CD4^+^ T cells. However, statistical significance was not reached for VOR and PEP005 at 6 hours. This could be due to the limited number of participants evaluated. Due to limited cell availability from PLWH donors, not all LRAs could be tested across all individuals, and therefore, only a subset of LRAs is shown in this analysis. VOR treatment was performed in resting CD4^+^ T cells using samples from a parallel study; responses were comparable with those observed in total CD4^+^ T cells.

### Correlation between ESR1 gene expression and ERα protein level downregulation in response to LRAs.

We next assessed the effect of *ESR1* gene expression downregulation by LRAs on ERα protein levels using Western blot analysis. As shown in Figure 2D, we observed a significant decrease in ERα protein levels after 6 hours of treatment with PMAi and after 24 hours of treatment with PMAi, ROM, and PEP005.

We further investigated the temporal relationship between *ESR1* mRNA downregulation and ERα protein level changes (Figure 2, B and D). Interestingly, a temporal delay was observed, where mRNA downregulation at 6 hours resulted in a more pronounced decrease in protein levels at the 24-hour time point. Significant mRNA downregulation at 6 hours following PMAi treatment led to the highest observed protein level decrease at 24 hours (0.37-fold change, *P* < 0.001). Similarly, the induction of mRNA downregulation by PEP005 at 6 hours resulted in a significant protein level decrease at 24 hours (0.57-fold change, *P* < 0.001). On the other hand, ROM, which demonstrated the highest downregulation at the 24-hour time point, results in only a minimal decrease at the same time point, likely as a result of a temporal lag in the decrease of protein synthesis as less transcripts become available for the translation machinery.

### Absence of ERα degradation by potent ERα degraders in CD4^+^ T cells.

**Figure 3:**
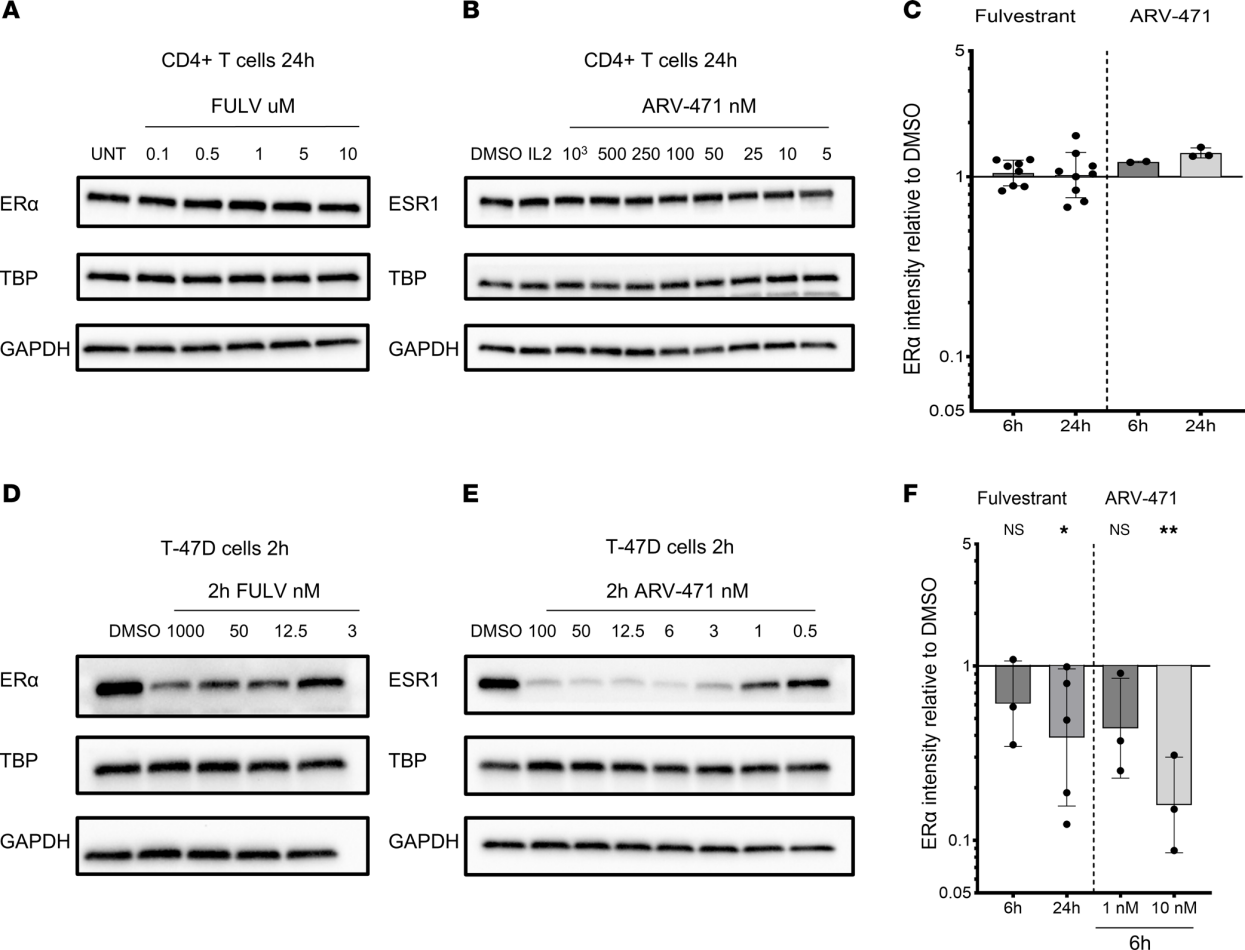
Comparison ERα degradation in CD4^+^ T cells vs T-47D. (**A** and **B**) Representative WB of ERα total protein from CD4^+^ T cells of seronegative donors after 24 hours of Fulvestrant or ARV-471 treatment at indicated concentrations. (**C**) Quantification of ERα total protein expression from CD4^+^ T cells after 6 or 24 hours of Fulvestrant (1 μM) treatment from *n* = 8 donors or ARV-471 treatment from *n* = 3 donors. (**D** and **E**) Representative WB of ERα total protein from T-47D cells after Fulvestrant or ARV-471 treatment at indicated concentrations at 2 hours post-treatment. (**F**) Quantification of ERα total protein from T-47D cells after Fulvestrant (1 μM) treatment 6 hours (*n* = 3) and 24 hours (*n* = 5) time points or after 6 hours ARV-471 treatment (*n* = 3) at 1 nM and 10 nM. Each point represents an individual sample. Geometric mean with geometric SD shown for **C** and **F**. *P < 0.05; **P < 0.005. One-sample *t* test was performed by comparing each treatment to the theoretical mean at the same time point.

To gain a better understanding of the relationship of the downregulation of the estrogen receptor and reactivation of HIV by LRAs, as well as the effect of this phenotype on immune function, we endeavored to use traditional tools to modulate ERα in CD4^+^ T cells. Selective estrogen receptor modulators (SERM) and selective estrogen receptor degraders (SERD) have shown promise in selectively targeting and degrading ERα protein in the breast cancer field. In this study, we evaluated ERα protein levels in CD4^+^ T cells exposed to Fulvestrant (FULV) a well-known SERD and ARV-471 (vepdegestrant), a PROTAC protein degrader, selective for ERα (21). Surprisingly, we did not observe any significant decrease in ERα protein levels after treatment with FULV at different concentrations and time points (Figure 3, A and C). Similarly, treatment with ARV-471 did not lead to significant ERα protein degradation in CD4^+^ T cells, even at higher doses and longer exposure times (Figure 3, B and C).

In contrast, as shown in Figure 3, D–F, consistent with previous findings (21), both FULV and ARV-471 successfully decreased ERα protein levels in T-47D breast cancer cells, demonstrating their efficacy as ERα degraders. Furthermore, we observed a dose- and time-dependent degradation of ERα in T-47D cells (Figure 3, D–F).

To confirm the degradation mechanisms in breast cancer cells, we examined *ESR1* gene expression in T-47D cells upon treatment with ERα selective modulators and degraders. After treatment of cells with these compounds, minor, nonsignificant changes in *ESR1* mRNA expression were observed (Supplemental Figure 2A), confirming that the decrease in ERα protein was directly related to targeting the protein for degradation and not a consequence of gene expression decrease. Similarly, in CD4^+^ T cells, we confirm that ERα modulators do not have an effect on *ESR1* expression (Supplemental Figure 2B). Of note, no sex differences were observed in these outcomes (data not shown).

Collectively, these results suggest a differential protein regulation in CD4^+^ T cells compared with breast cancer cells (T-47D cells), where ERα modulators were originally developed and extensively studied. The differential response of CD4^+^ T cells to ERα degraders raises intriguing questions regarding the regulation of ERα protein in immune cells and the ERα pathway involved in cell differentiation and cytokine modulation.

### Absence of ERα protein nuclear localization and translocation in CD4^+^ T cells.

**Figure 4:**
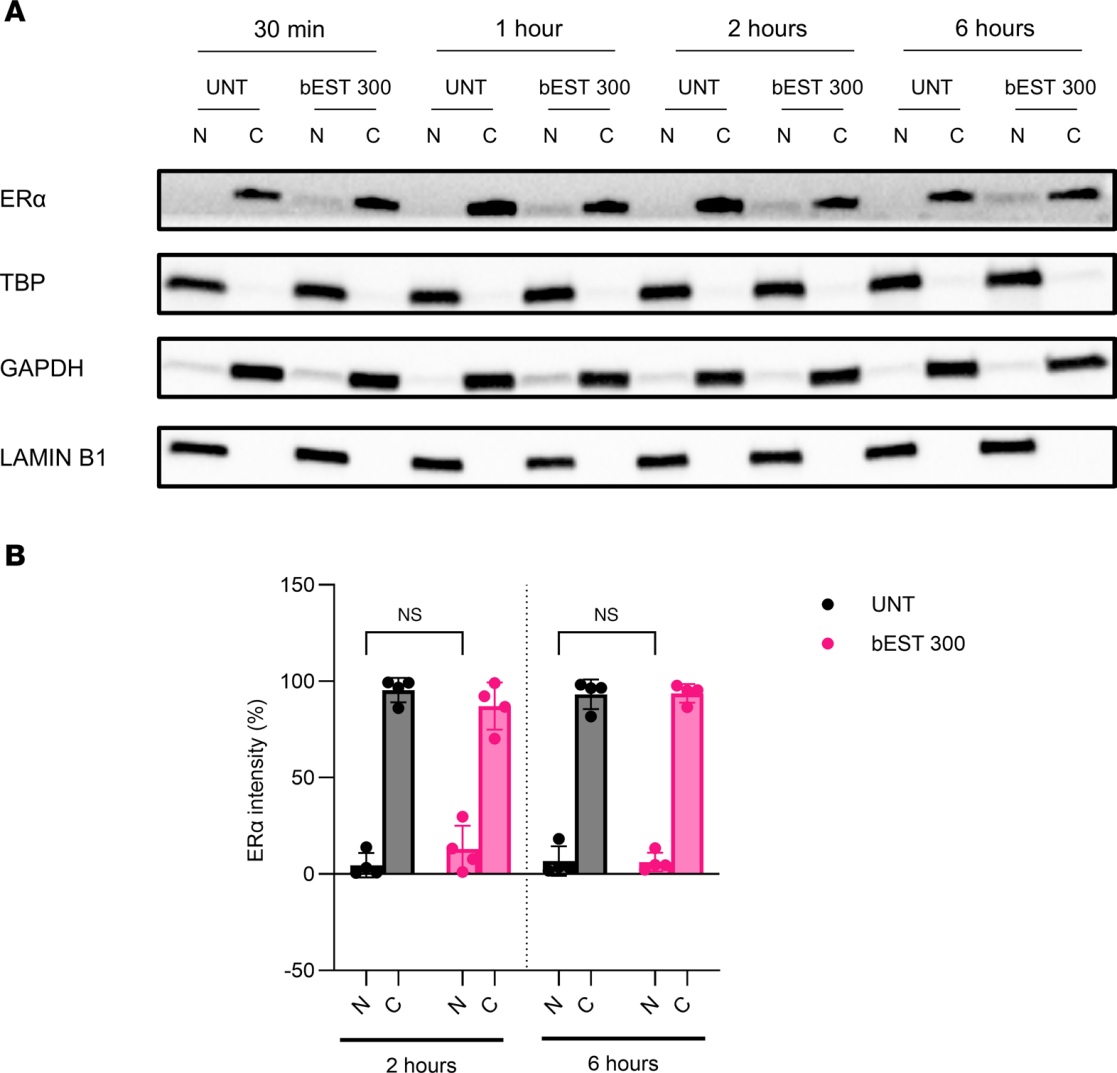
Absence of ERα protein nuclear localization and translocation in CD4^+^ T cells. (**A**) Representative WB of ERα nuclear (N) and cytoplasmic (C) protein fraction from CD4^+^ T cells of seronegative donors after β-estradiol (300 pg/mL) treatment from 30 minutes to 6 hours. (**B**) Quantification of ERα protein expression in the N and C compartment from *n* = 4 donors after 2 hour and 6 hours in untreated conditions and treatment with β-estradiol (300 pg/mL). Each point represents an individual sample. Mean ± SD shown for **B**. Statistical significance was calculated using Mann-Whitney *U* test between nucleus protein quantification with and without treatment at the same time point.

Given our unexpected results with the ERα degraders in CD4^+^ T cells, we next investigated the subcellular localization and translocation of ERα in CD4^+^ T cells, along with its modulation by β-estradiol (βEST) treatment. Estrogen receptor signaling is complex and occurs through both ligand-dependent and ligand-independent pathways. Ligand-dependent signaling is initiated by the binding of endogenous or synthetic estrogen to the ER receptor in the cytosol, triggering a conformational change leading to the translocation of the receptor to the nucleus where it binds to the estrogen-response element (ERE) of estrogen-responsive genes to regulate transcription. We therefore exposed CD4^+^ T cells to physiologically relevant concentrations of βEST and assessed nuclear translocation of ERα. Surprisingly, we observed a prevalent cytoplasmic localization of ERα (95%) in CD4^+^ T cells, indicating a minimal presence in the nucleus. Furthermore, despite varying concentrations and treatment durations of βEST, no significant nuclear translocation of ERα was induced (Figure 4, A and B). This was true even for brief 30-minute (Figure 4A) or extended 24-hour exposure to βEST (data not shown).

To validate these findings and evaluate the bioactive properties of βEST, parallel experiments were conducted in the breast cancer cell line T-47D. In contrast to CD4^+^ T cells, untreated T-47D cells demonstrated predominant nuclear localization of ERα (70%). Notably, βEST treatment induced significant translocation of the ERα from the cytoplasm to the nucleus (Supplemental Figure 3, A and B). Additionally, βEST-treated T-47D cells exhibited significant enhanced metabolic activity (Supplemental Figure 3C) and duplication rate (Supplemental Figure 3D) compared with untreated conditions, confirming the bioactivity of the βEST used in this study. Similarly, pretreatment of CD4 T cells with βEST prior to HIV infection resulted in a decrease in HIV p24 production compared with untreated control cells as previously reported (24), confirming the biological activity of our stock of βEST in primary cells (Supplemental Figure 4). These results highlight the differential modulation of ERα in CD4^+^ T cells and breast cancer cells, suggesting distinct regulatory mechanisms requiring further investigations.

## Discussion

Given the financial, social and psychophysiological cost of persistent HIV infection, defining an HIV cure remains a priority. Therefore, understanding factors that may influence the efficacy of cure interventions being developed is crucially important. Differences in the levels and the physiological responses to sex hormones such as estrogen can affect HIV transcription and immune function. This is likely in turn to influence outcomes of curative therapies such as those employing latency reversal followed by immune augmentation to clear HIV-infected cells. In this study, we sought to understand the effect of LRAs on ERα signaling in CD4^+^ T cells obtained from individuals who are seronegative, and men and women living with HIV durably suppressed on ART. Our findings suggest a strong interaction between LRAs commonly used in the shock-and-kill HIV cure strategy and ERα on immune cells. Notably, we observed that common LRAs being investigated for cure approaches significantly downregulated *ESR1* mRNA leading to a decrease in ERα protein production. However, interestingly, directly targeting ERα by SERM, SERD, and ER-specific PROTAC degraders does not appear to be a viable strategy to modulate ERα protein levels in T cells. Furthermore, we also demonstrate that, in CD4^+^ T cells, ERα localization in response to exogenous estrogen is different than what has previously been demonstrated in breast cancer cells. In CD4^+^ T cells, ERα remains localized to the cytoplasm, with marginal nuclear translocation following exposure to exogenous estrogen.

The importance of estrogen in modulating HIV infection has previously been reported (8, 9, 25–28). There are several types of estrogens secreted endogenously across the lifespan. These include estrone (dominant form in postmenopausal women), estradiol (primary form during reproductive age), and estetrol and estriol (secreted during pregnancy). 27-Hydroxycholesterol (27HC), a cholesterol metabolite with weak estrogenic activity, is sometimes considered another form of estrogen, but less is known about its physiological relevance in this context. Of these, estradiol is the most potent form of estrogen and has the highest affinity for the estrogen receptors (10). Estrogen levels vary across the menstrual cycle in women, ranging from 30 pg/mL to 400 pg/mL, with peak levels reaching 400 pg/mL during the secretory/follicular stage of the menstrual cycle in some individuals. By comparison, in men levels range from 10 to 50 pg/mL. During menopause, estrogen levels decrease, and they range from < 10 pg/mL to 30 pg/mL in postmenopausal women (29–31). Interestingly 1 study detected lower plasma viral load in untreated women during the ovulatory phase of the menstrual cycle (high estrogen environment), suggesting that estrogen creates a suppressive environment for HIV replication (32). More recent investigations into the role of estrogen in HIV transcription have shown that in the presence of estrogen, the transcription factor, β-catenin can complex with ERα at the HIV LTR to restrict transcription (8). Furthermore, estrogen can blunt TCR reactivation of latent HIV in CD4^+^ T cells isolated from aviremic PLWH ex vivo (9). It is therefore possible that downregulation of ERα by LRAs contributes to the increase in HIV transcription reported following latency reversal.

In addition to its effect on HIV transcription, estrogen signaling through its receptor can modulate the immune response, either stimulating or suppressing immune function contextually and depending on the cell type (10). Signaling through the estrogen receptor can both negatively and positively influence diverse immune functions, including but not limited to cytokine production, T cell proliferation, monocyte differentiation, monocyte/macrophage chemotaxis, macrophage polarization, and NK cell function (10, 33). Although HIV primarily infects CD4^+^ T cells, ERα is expressed in other immune subsets within PBMCs. Dissecting ERα expression and function across other cell types could provide additional insight into estrogen signaling in the immune system and its potential effect on HIV biology and warrants further study. Moreover, even within the CD4^+^ T cell compartment, distinct functional subsets (e.g., Th1, Th2, Th17, Treg, Tfh) may differ in ERα expression or responsiveness to estrogen, which could contribute to heterogeneous effects on HIV latency and immune activation. Thus, while the downregulation of ERα by LRAs is likely to positively affect HIV transcription, the implications for immune modulation are significant and merit further interrogation. The exact mechanisms involved in LRA downregulation of ERα are unclear. ER function can be influenced by different coregulators, and modulation of these coregulators by LRAs could have a secondary effect on the estrogen receptor (34). Whatever mechanisms are involved share a common pathway, as exposure to various LRAs resulted in some degree of downregulation of the receptor. As LRAs are increasingly being tested in the clinic in conjunction with immune boosting interventions, it will be important to further understand the effect of ERα modulation by LRAs on immune response.

As mentioned previously, signaling through the estrogen receptor is a complex process and can involve both canonical (genomic) and noncanonical (nongenomic) pathways; it involves both ligand-dependent and ligand-independent methods. Ligand-dependent signaling triggers genomic effects of estrogen (e.g., changes at the level of gene transcription) and is initiated by the binding of endogenous or synthetic estrogen to the ER receptor in the cytosol, prompting dimerization of the receptor leading to its translocation to the nucleus where it binds to ERE on regulatory regions of estrogen-responsive genes to regulate transcription (35). Ligand-activated ER can also interact with other transcription factors such as AP1, SP1, and NF-κB at their consensus DNA binding sites to indirectly regulate AP1, SP1, and NF-κB–dependent transcription (33, 35, 36). Nongenomic effects are triggered when estrogen binds to plasma membrane ER variants or the G protein-coupled estrogen receptor (GPER1) leading to conformational changes in the receptors and activation of several signaling pathways, including the phosphatidylinositol-3-kinase (PI3K), Protein kinase A (PKA), and mitogen-activated protein kinase (MAPK) pathways, resulting in modulation of downstream target genes (35, 37). Lastly, in the ligand-independent signaling pathway, in the absence of estrogen, ER is activated following phosphorylation by various protein kinases leading to either direct ERE binding or interaction with other transcription factors (36). Our study focused on the classical or canonical estrogen signaling pathway. An interesting finding of this study is that ERα was located primarily in the cytoplasm in CD4^+^ T cells and failed to translocate to the nucleus upon exposure to estrogen in comparison to the breast cancer cell line T-47D. Furthermore, we were not able to modulate ERα in CD4^+^ T cells using traditional (SERM and SERD) and innovative (PROTAC) methods used in breast cancer cell lines. There are several implications to this observation. It is possible that estrogen signaling in T cells occurs predominantly through noncanonical, extranuclear pathways, which have been linked to rapid activation of MAPK and NF-κB cascades rather than direct transcriptional regulation (33, 35, 36). Such nongenomic signaling may influence HIV latency by modulating T cell activation and HIV transcription indirectly.

Both ERα and ERβ are present in multiple isoforms generated through alternative splicing or differential promoter usage. There are 3 main variants of ERα, with different molecular weights, ERα36, ERα46, and ERα66. Of these, ERα66 is the canonical receptor, whereas ERα46 lacks the N-terminal AF-1 transcriptional activation domain, and ERα36 lacks both AF-1 and the AF-2 C-terminal transcriptional activation domains but retains the ability to bind DNA (38–40). ERα36 resides predominantly in the cytoplasm and plasma membrane, and while it cannot independently activate classical estrogen responsive gene transcription, it can interact with and negatively regulates the transcriptional activity of ERα66 and competes with ERα66 for binding EREs (41). It can also modulate transcription through nongenomic pathways (41). The antibody we used in our study primarily recognizes canonical ERα (ERα66). Thus, it is possible that the SERM and SERD used in this study modulated the noncanonical ER variants but were missed in our assays. Furthermore, it is possible that estrogen signaling through the membrane receptor GPER1 plays a significant role in CD4^+^ T cell function, and this was not assessed in our study. Interestingly, GPER1 can interact with ERα36 at the plasma membrane to modulate transcription through activation of signaling pathways such as MAPK/ERK and PI3K/AKT (42, 43). However, while most of these functions have been characterized in oncology, the role of ER isoforms and GPER1 in immune cells remains incompletely understood and requires further investigation. Lastly, as previously reported (34, 44, 45), cell type–specific regulation of estrogen receptors and responses to their modulators, as well as potentially novel or as-yet-uncharacterized estrogen receptor variants in CD4^+^ T cells, may clarify how estrogen influences T cell behavior beyond what is accounted for by classical ER signaling and might explain our findings.

Despite the limitations of our study, our results suggest that the control of canonical signaling in CD4^+^ T cells is different than what is reported in cancer cells and may involve the activity of yet-to-be-identified critical players and merits further investigation. Furthermore, our findings highlight an important unanswered question: whether LRAs downregulate ERα in vivo and what implications this may have for HIV transcription and immune signaling. Given that multiple clinical trials have evaluated the in vivo efficacy of LRAs, it would be of interest to define how these agents influence estrogen receptors in vivo.

## Methods

### Sex as a biological variable.

Our study examined male and female participants, and similar findings are reported for both sexes.

### Study participants.

Durably suppressed on ART (HIV-1 RNA < 50 copies/mL for at least 12 months) male and female PLWH were recruited through the UNC Global HIV Prevention and Treatment Clinical Trials Unit and the UNC Center for AIDS Research HIV Clinical Cohort. Whole blood or PBMC from HIV seronegative donors were commercially sourced (New York Blood Center, New York, New York, USA, and Stemcell Technologies, respectively). Genetic sex was determined by qPCR detection of amelogenin X-linked (AMELX), amelogenin Y-linked (AMELY) and sex-determining region Y (SRY) genes as previously published (46, 47).

### Cell isolation and treatment conditions.

PBMCs, CD4^+^ T cells, and resting CD4^+^ T cells were obtained and cultured as previously described (48). Of note, PBMC and CD4^+^ T cells were cultured in hormone-free media condition, defined as IMDM without phenol red and with charcoal-stripped fetal bovine serum (CS-FBS). Resting CD4^+^ T cells were cultured in regular media (with phenol red and with regular FBS) as part of a different study being conducted in parallel. No differences in *ESR1* modulation were observed in cultures containing regular media and hormone-free media.

Treatment with LRAs was performed for 6 and 24 hours at the following concentrations: PHA/IL-2 (2 μg/mL plus 100 U/mL), PMA plus ionomycin (16 nM plus 0.5 μM), VOR (340 nM), ROM (20 nM), PEP005 (12 nM), AZD-5582 (100 nM), iBET-151 (1,000 nM), STINGa (100 nM), or complete medium plus 0.1% DMSO (37°C, 5% CO_2_). Except for PHA (Remel, Thermo Fisher) and STINGa (22), all LRAs were procured from Selleckchem. The media for all conditions (except PHA/IL-2) were also supplemented with 5 U/mL of IL-2. For AZD and STINGa stimulations, the medium containing the drugs was replaced with fresh medium without LRAs after 3 and 6 hours, respectively. All the LRA concentrations used in this study are values reported in the literature (23, 49–51).

Estrogen receptor modulators were used at 300 pg/mL for βEST (Sigma-Aldrich), 1 μM for Tamoxifen (Selleckchem). Ranges of concentrations from 0.1 μM to 10 μM for FULV (Selleckchem) and from 0.5 nM to 1 μM for ARV-471 (MCE) were used. Specific time points evaluated are as indicated in the results section and range from 30 minutes to 24 hours.

For each condition, at each time point, at least 2 replicate wells were used for gene expression experiments.

### CD4 T cell infection.

CD4^+^ T cells were isolated from PBMC obtained from HIV-seronegative buffy coats (New York Blood). CD4^+^ T cells were activated with 2 μg/mL PHA + 60 U/mL IL-2 and treated with βEST (300 pg/mL and 600 pg/mL) for 3 days. Controls were activated with the above concentration of PHA/IL-2 but not exposed to βEST. Cells were infected with the lab-adapted HIV strain, JRCSF (NIH HIV Reagent Program) at an m.o.i. of approximately 1 for 2 hours at 37°C under 5% CO^2^, extensively washed to remove unbound virus and the infection was allowed to proceed for 7 days. Supernatant was collected and HIV p24 measured using the ABL p24 ELISA system following the manufacturer’s protocol (ABL, Rockville, MD). HIV p24 was quantitated using a SpectraMax M3 Microplate reader (Molecular Devices).

### Breast cancer T-47D cell line culture and treatment conditions.

T-47D breast cancer cell line (ATCC) was maintained in RPMI 1640 supplemented with 10% heat-inactivated fetal bovine serum (FBS), 100 U/mL penicillin, and 100 μg/mL streptomycin (37°C, 5% CO_2_). For βEST and ERα modulator treatment experiments, cells were deprived of steroid hormone by placement in hormone free media, as previously mentioned, for 5–6 days prior to performing the treatment experiments. For total, nuclear, and cytoplasmatic protein extraction, cells were plated at 6 × 10^5^ million cells/cm^2^. For the metabolic assay, cells were plated in 96-well plates at 1 × 10^4^ million cells/ cm^2^ in 100 μL media. For proliferation induction by βEST, cells were plated at 2 × 10^4^ million cells/ cm^2^. After overnight incubation, cells were treated with βEST, FULV, or ARV-471 at concentrations and time points described in the result section. For metabolic activity assays and proliferation induction by βEST, media with and without βEST were changed every 2 days. The PrestoBlue Cell Viability Reagent (Thermo A13261) assay was performed following the manufacturer’s instructions. Eight technical replicates per condition were performed. PrestoBlue reagent was added at 10% of the volume in the well immediately (day 0) and at days 2, 4, and 5. The cells were incubated with the PrestoBlue reagent at 37°C, 5% CO_2_ in the dark, for 2 hours and fluorescence was detected using the SpectraMax M3 plate reader (Molecular Devices). For βEST proliferation induction experiments, cells were harvested and counted using at least 3 independent readings for each condition.

### Gene expression.

RNA extraction, quantification, cDNA synthesis, quantitative real-time PCR, and data analysis were performed as previously reported (48). Primers/probes were obtained from Integrated DNA Technologies (IDT). RPL27 and TBP were used as reference genes; detailed information, previous validation and gene of interest expression profile calculations are reported elsewhere (48). *ESR1* primers/probes used in this study are as followed: F5′- GAACCGAGATGATGTAGCCA-3′;R5′-GTTTGCTCCTAACTTGCTCTTG-3′; /56-FAM/AGATCTCCA/ZEN/CCATGCCCTCTACACA/3IABkFQ/ (IDT ID Hs.PT.58.14846478).

### Protein quantification.

For total protein extraction, cell lysis was performed from a minimum of 2 × 10^6^ cells in a 1X RIPA buffer (Cell Signaling, 9806) supplemented with 1X Halt Protease Inhibitor Cocktail, EDTA-Free (Thermo Fisher Scientific, 87785) and 4 μL/mL Pierce Universal Nuclease for Cell Lysis (Thermo Fisher Scientific, 88701) for 30 minutes on ice. Following lysis, cellular debris were pelleted by centrifugation at 20,000*g* for 10 minutes at 4°C, and the protein-containing supernatants were recovered for subsequent quantification.

For cytoplasmic and nuclear fractions, a minimum of 5 × 10^6^ cells were used. NE-PER Nuclear and Cytoplasmic Extraction Reagents (Thermo Fisher Scientific, 78833) were used to isolate cytoplasm protein per the manufacturer’s instructions with the following modification for the nuclear protein extraction. Following cytoplasmic supernatants collection, nuclear proteins were extracted as previously described for total protein extraction with an incubation on ice for 40 minutes.

Protein concentrations were measured by the Pierce BCA Protein Assay Kit (Thermo Fisher Scientific, 23225) according to manufacturer’s instructions with incubation time of 30 minutes for total protein and 2 hours for cytoplasmic and nuclear fractions. In total, 5 μg of protein was separated on 4%–20% Tris-glycine acrylamide gel (TGX gels, Bio-Rad, 5671094) and transferred unto Immuno-Blot PVDF membranes via semidry transfer using the Trans-Blot Turbo System (Bio-Rad). Membranes were blocked with 1X tris-buffered saline (TBS) containing 5% milk for at least 30 minutes. Primary antibodies were diluted at 1:10,000 in 1X TBS containing 0.5% Tween-20 (TBST) and 5% milk and incubated overnight at 4°C on a rocker. Membranes were washed 3 times in TBST and then incubated with appropriate HRP-conjugated secondary antibody (Life Technologies) at 1:100,000 dilution in TBST/milk for 1 hour at room temperature. Membranes were washed 3 times with TBST, before being developed for ERα (Abcam, ab108398) and TBP (Abcam ab220788) using SuperSignal West Atto Ultimate Sensitivity Substrate (Thermo Fisher Scientific, A38555). GAPDH (Abcam, ab181602) and LAMIN B1 (Abcam, ab133741) were developed using SuperSignal West Dura Extended Duration Substrate (Thermo Fisher Scientific, 34075). Blots were imaged using the Bio-Rad Versadoc imager and analyzed using Image Lab software.

### Statistics.

Statistical analyses of gene expression data were conducted using GraphPad Prism 8 (version 8.4.2). Statistical significance was calculated using the appropriate statistical test for the data and is detailed in the figure legends. The following statistical tests were used: Mann-Whitney *U* test, Wilcoxon test 2-tailed, 1-sample *t* test (2-tailed), 2-tailed paired *t* test, and 2-way ANOVA, as appropriate. *P* < 0.05 was considered significant.

### Study approval.

All samples from PWH used in this study were collected under a protocol approved by the UNC Biomedical IRB. All participants provided written informed consent.

### Data availability.

Values for all data points in graphs are reported in the Supporting Data Values file.

## Author contributions

CC and NMA conceptualized the study and designed the experiments. CC, PK, AAL, KJL, PM, BA, and KSJ performed the experiments. CC, NMA, PK, AAL, and KJL analyzed the data. DMM and AMWT contributed to the design of the study and data interpretation, and they provided important intellectual content. CC and NMA wrote the original draft of the manuscript. All authors participated in revising the manuscript.

## Conflict of interest

The authors have declared that no conflict of interest exists.

## Funding support

- National Institutes of Health National Institute of Allergy and Infectious Diseases grant nos. NIH 1R56AI170226 to NMA.
- UM1-AI164567 (Collaboratory of AIDS Researchers for Eradication [CARE]) to DMM.
- U54-AI170792 (HIV Accessory and Regulatory Complexes [HARC]), PI, Krogan; Subgrant 13897SC to NMA.
- NIH P30AI50410 to University of North Carolina Center for AIDS Research (CFAR).

## Supplementary Material

Supplemental data

Unedited blot and gel images

Supporting data values

## Version 1 07/10/2026

In-Press Preview

## Version 2 08/10/2026

Electronic publication
