# Supplementary material for: Effect of ligands and HIV latency-reversing agents on estrogen receptor α in CD4+ T cells
Source: JCI Insight. 2026 Jul 2;11(15):e198415. doi: 10.1172/jci.insight.198415 (PMC13464009; doi:10.1172/jci.insight.198415)
Supplement: Supplementary file 1 [file jciinsight-11-198415-s291.pdf]

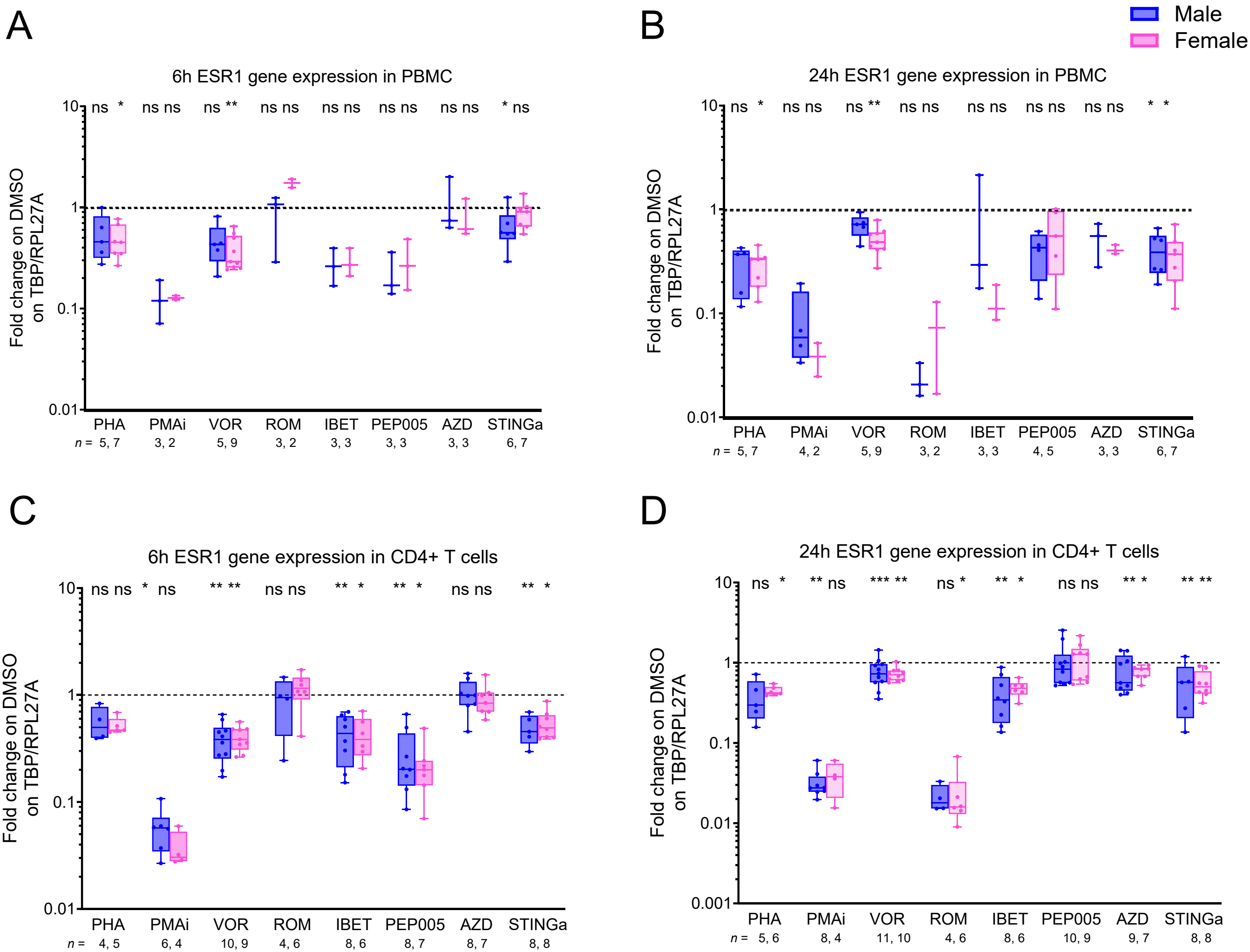

A
B
Male
Female
6h ESR1 gene expression in PBMC
24h ESR1 gene expression in PBMC
Fold change on DMSO on TBP/RPL27A
PHA PMAi VOR ROM IBET PEP005 AZD STINGa
n = 5, 7 3, 2 5, 9 3, 2 3, 3 3, 3 3, 3 6, 7
n = 5, 7 4, 2 5, 9 3, 2 3, 3 4, 5 3, 3 6, 7
C
D
6h ESR1 gene expression in CD4+ T cells
24h ESR1 gene expression in CD4+ T cells
n = 4, 5 6, 4 10, 9 4, 6 8, 6 8, 7 8, 7 8, 8
n = 5, 6 8, 4 11, 10 4, 6 8, 6 10, 9 9, 7 8, 8

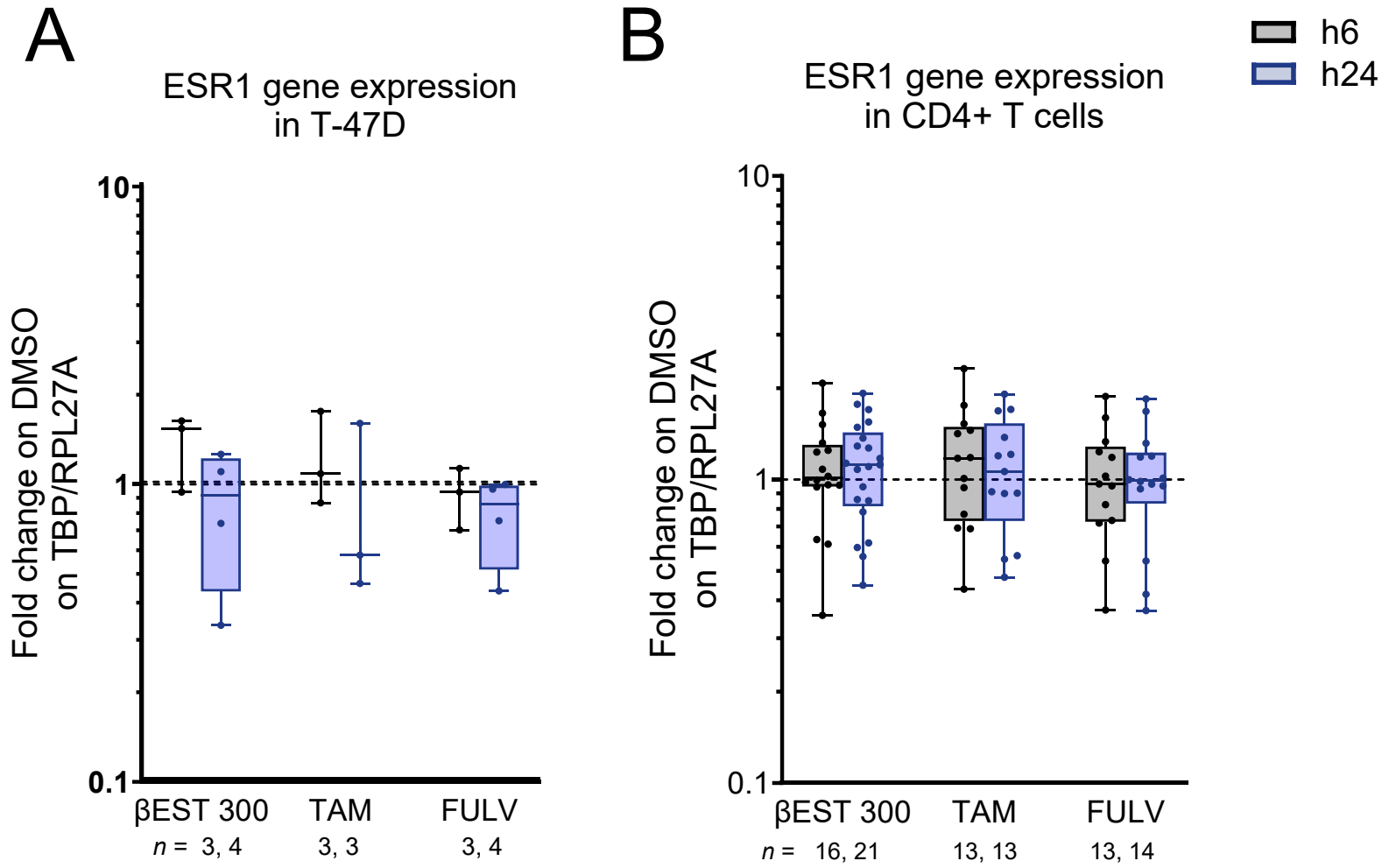

A
ESR1 gene expression
in T-47D
10
1
0.1
Fold change on DMSO
on TBP/RPL27A
βEST 300
TAM
FULV
n = 3, 4
3, 3
3, 4
B
ESR1 gene expression
in CD4+ T cells
10
1
0.1
Fold change on DMSO
on TBP/RPL27A
βEST 300
TAM
FULV
n = 16, 21
13, 13
13, 14
h6
h24

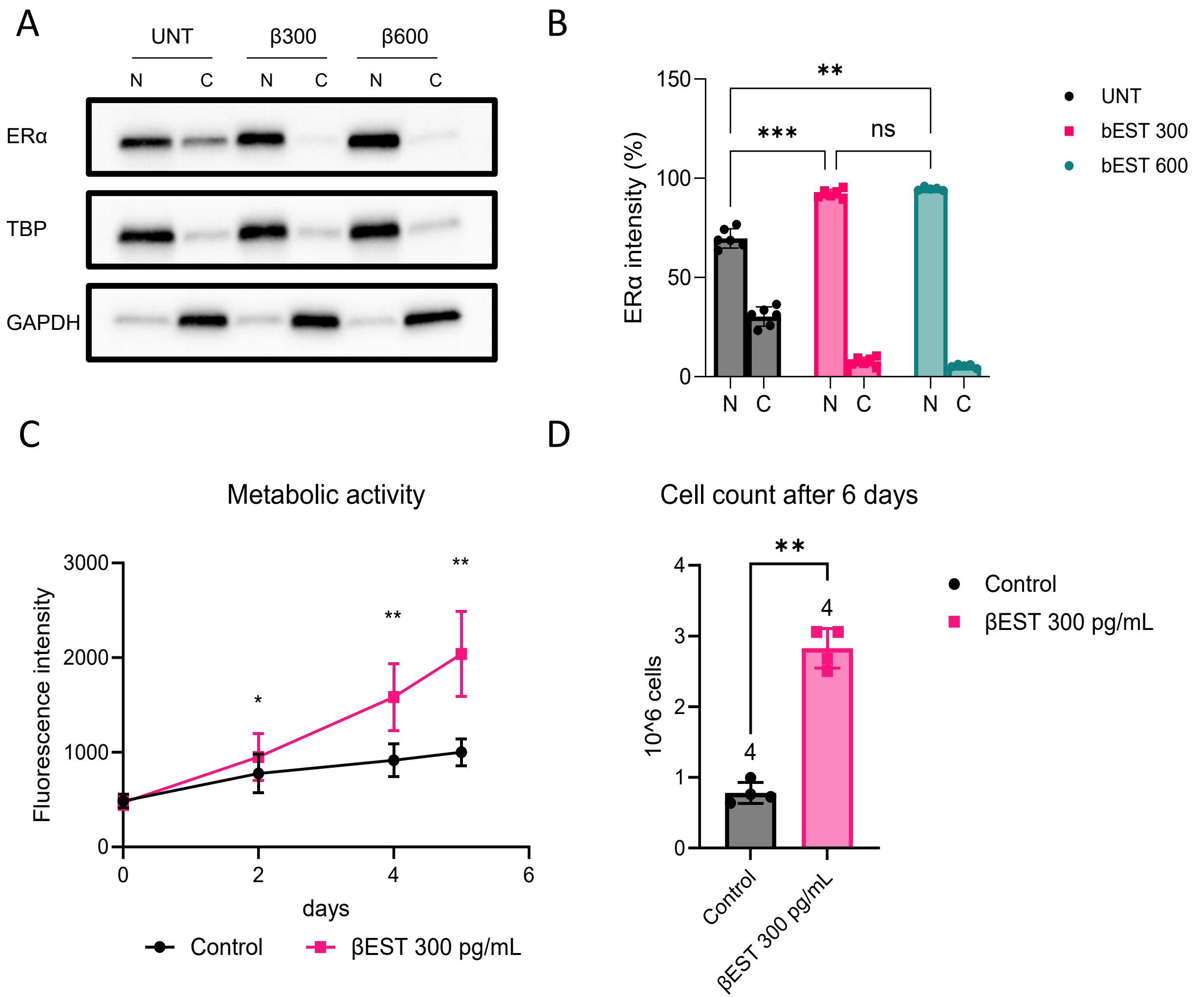
A
UNT
β300
β600
N
C
N
C
N
C
ERα
TBP
GAPDH
B
ERα intensity (%)
150
100
50
0
**
***
ns
N
C
N
C
N
C
UNT
bEST 300
bEST 600
C
Metabolic activity
Fluorescence intensity
3000
2000
1000
0
*
**
**
0
2
4
6
days
Control
βEST 300 pg/mL
D
Cell count after 6 days
10^6 cells
4
3
2
1
0
**
4
4
Control
βEST 300 pg/mL
Control
βEST 300 pg/mL

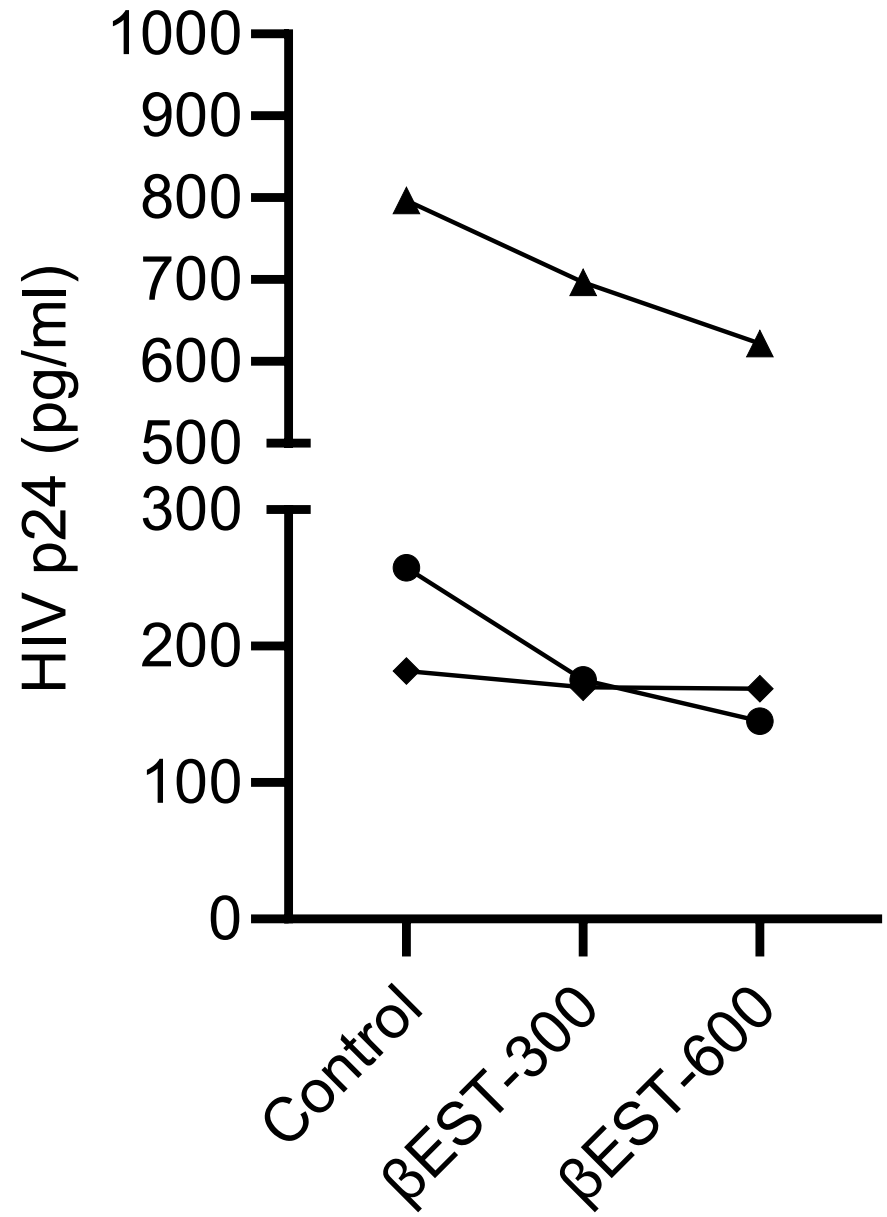
HIV p24 (pg/ml)
1000
900
800
700
600
500
300
200
100
0
Control
βEST-300
βEST-600
