# Supplementary material for: Effect of ligands and HIV latency-reversing agents on estrogen receptor α in CD4+ T cells
Source: JCI Insight. 2026 Jul 2;11(15):e198415. doi: 10.1172/jci.insight.198415 (PMC13464009; doi:10.1172/jci.insight.198415)

Full unedited blot/gel for Figure 3A

ECL exposure (bands)

Visible-light image (marker)

CD4+ T cells 24h

FULV uM

UNT 0.1 0.5 1 5 10

ERα

TBP

GAPDH

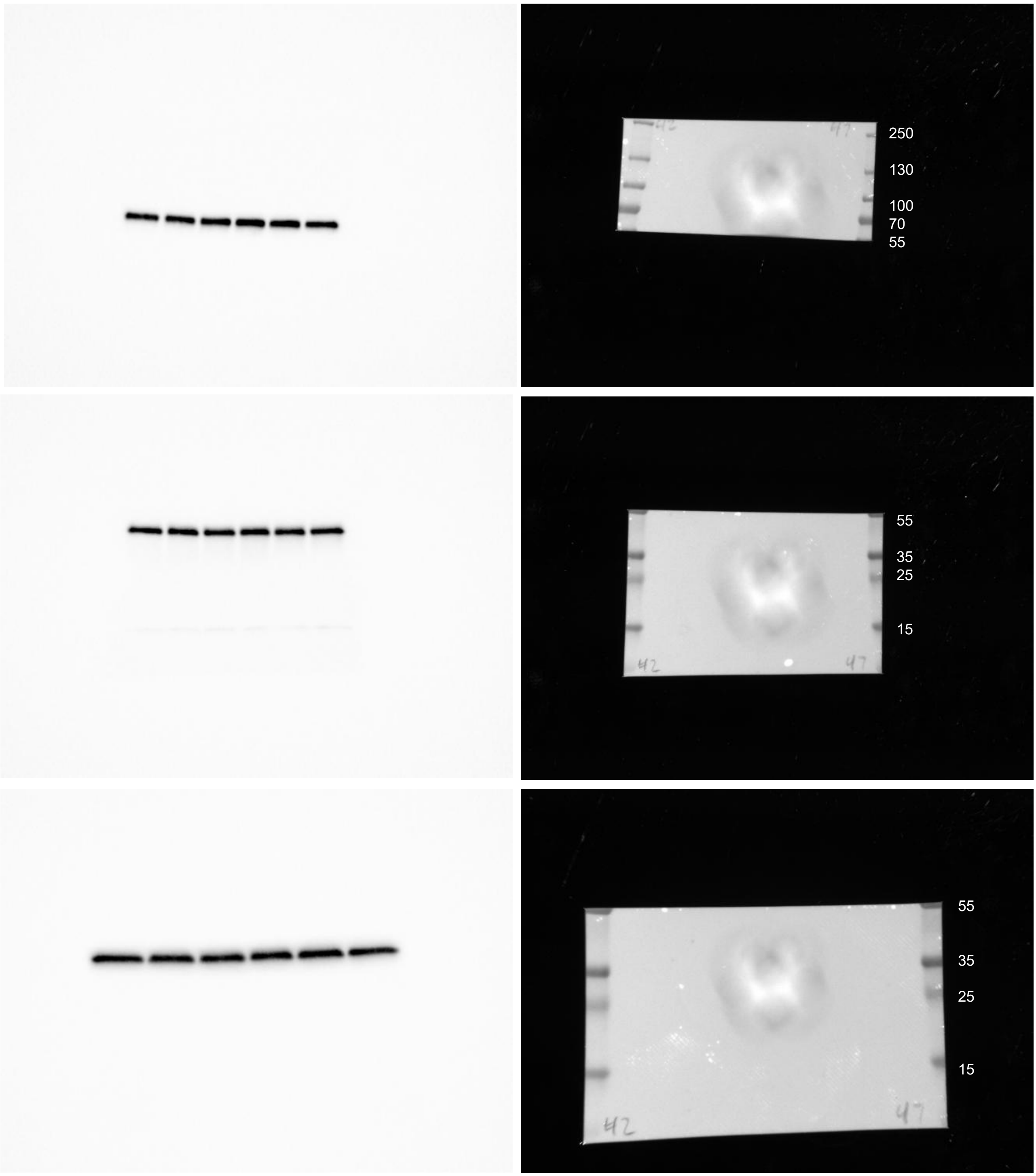

Full unedited blot/gel for Figure 3B

ECL exposure (bands) Visible-light image (marker)

CD4+ T cells 24h

ARV-471 nM

DMSO IL2 10^3 500 250 100 50 25 10 5

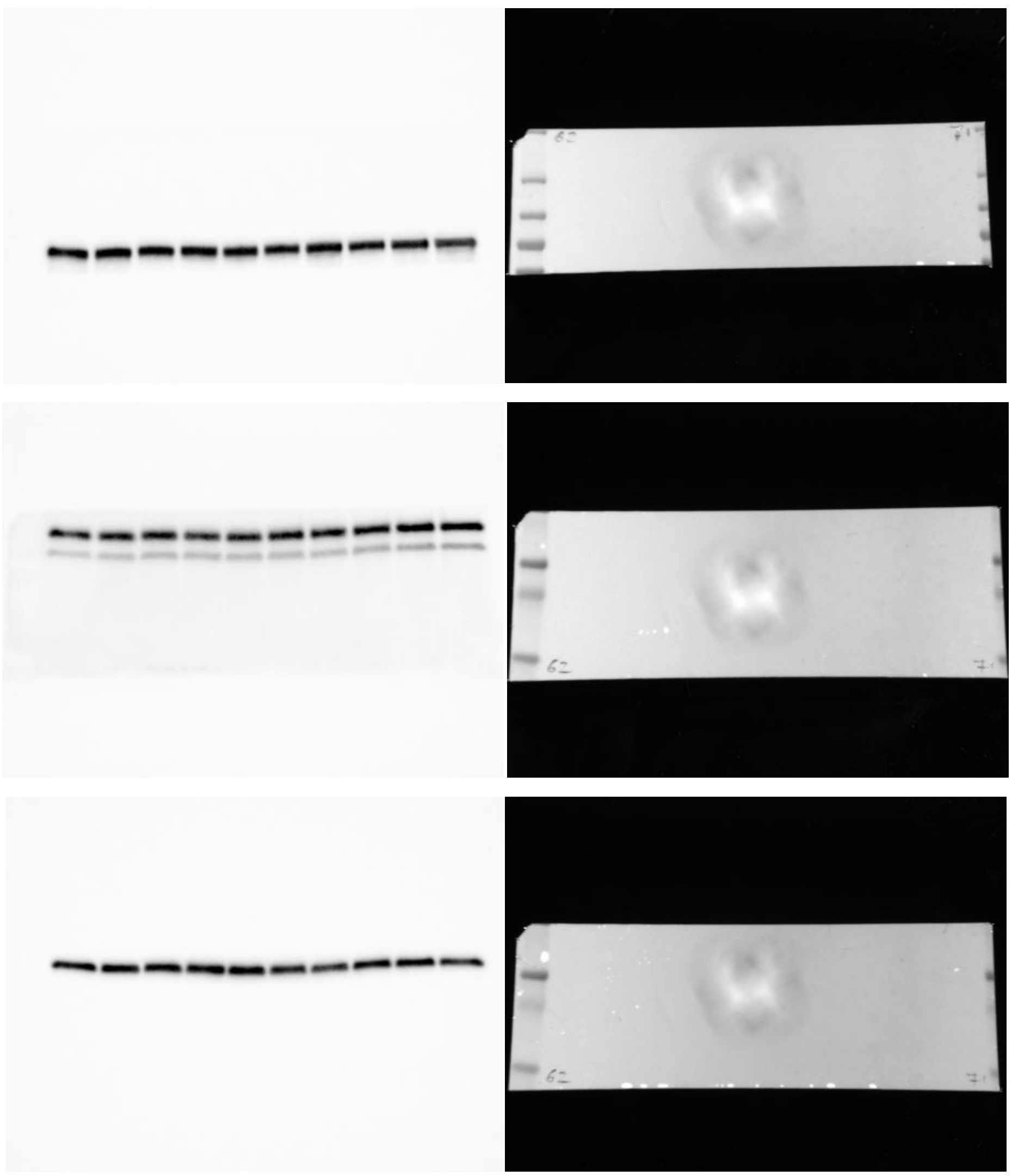

Full unedited blot/gel for Figure 3D

ECL exposure (bands) Visible-light image (marker)

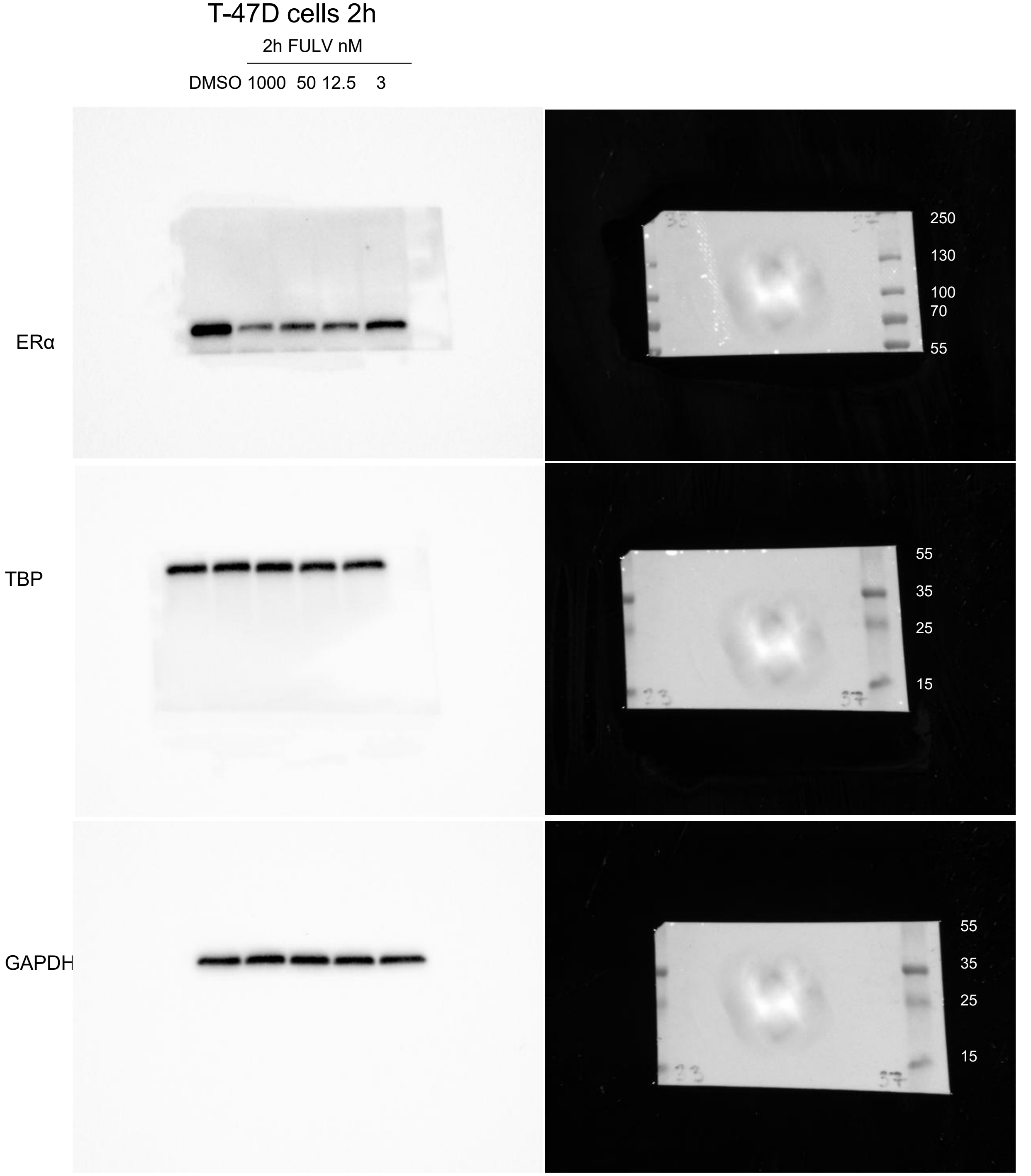

Full unedited blot/gel for Figure 3E

ECL exposure (bands) Visible-light image (marker)

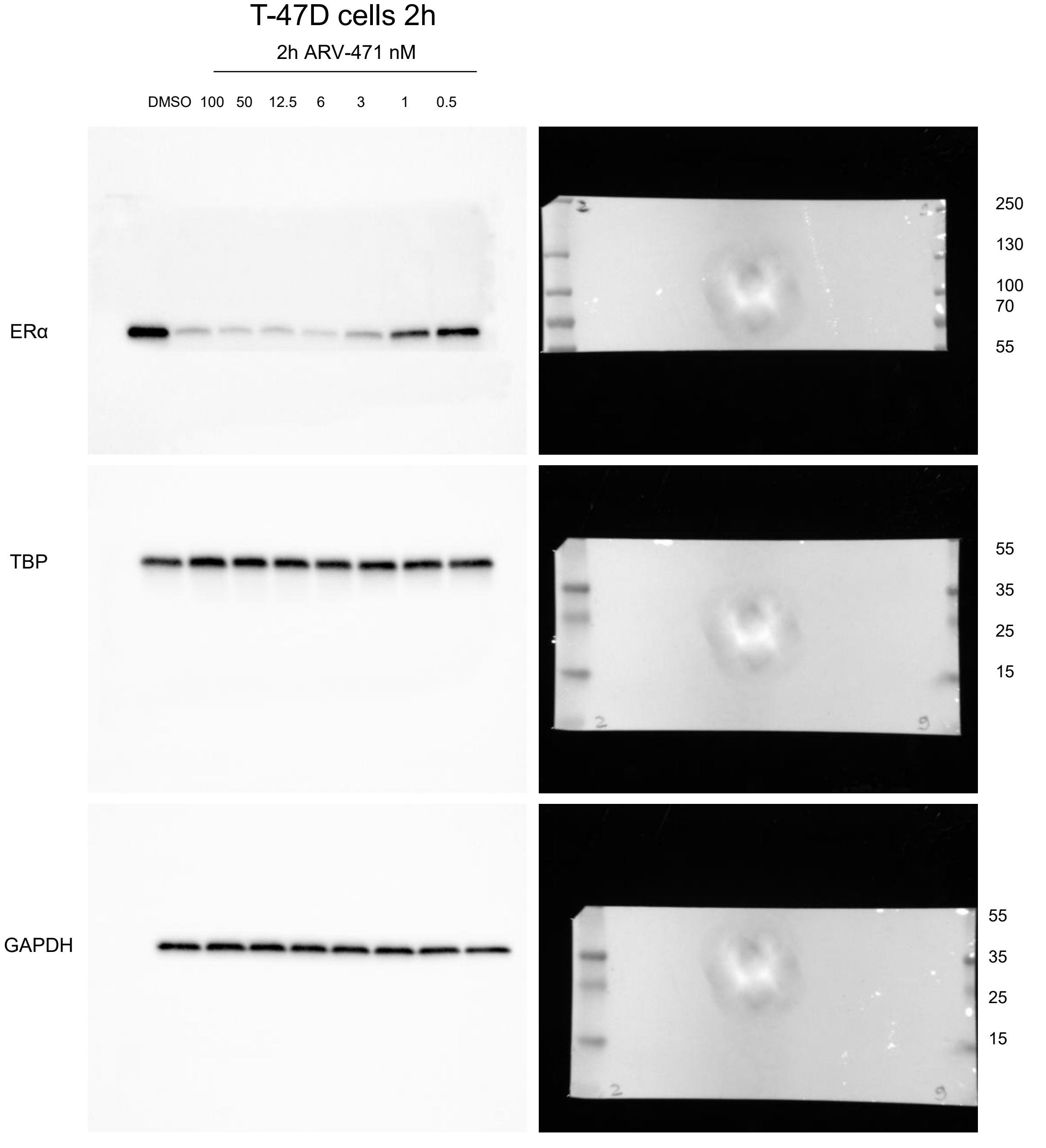

Full unedited blot/gel for Figure 4A

ECL exposure (bands)

Visible-light image (marker)

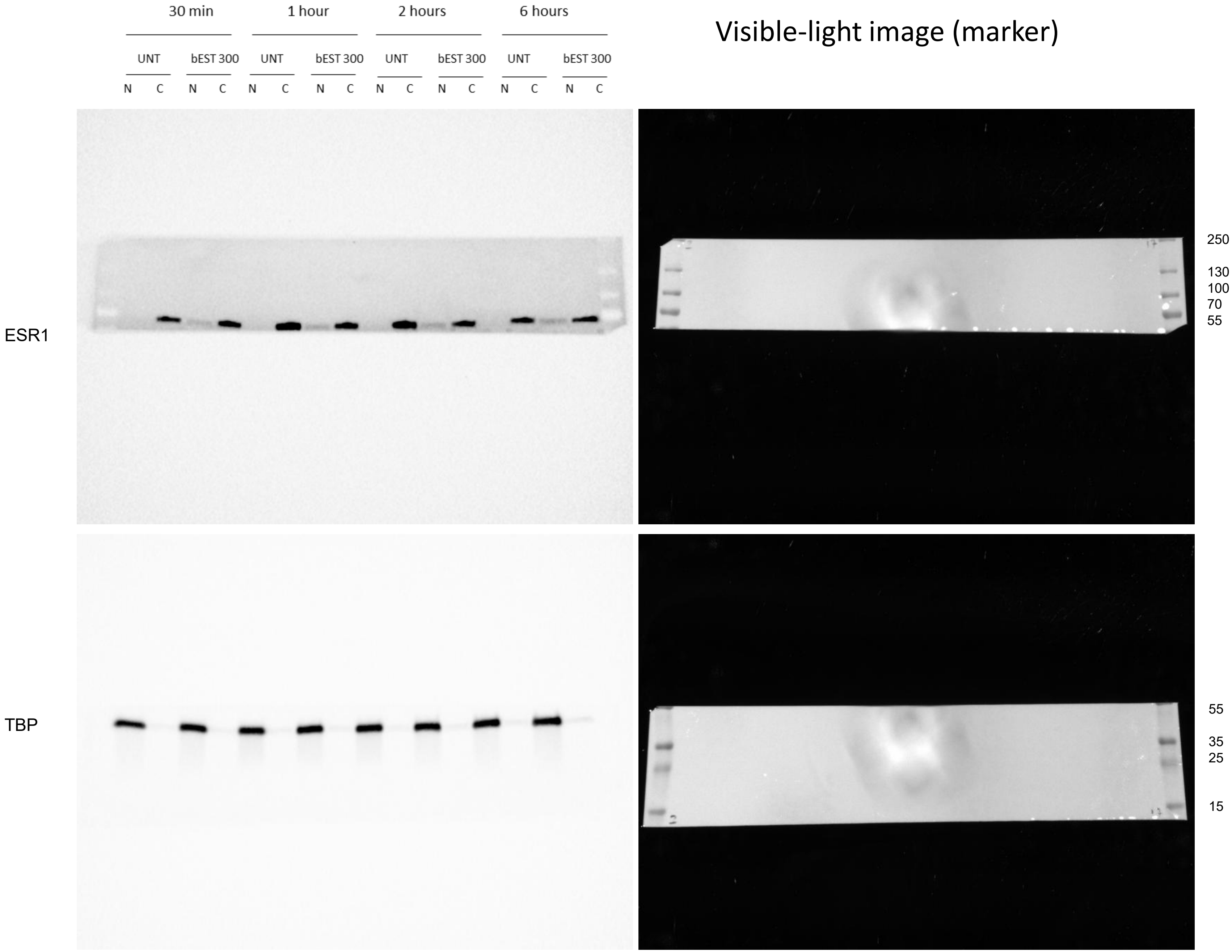

Full unedited blot/gel for Figure 4A

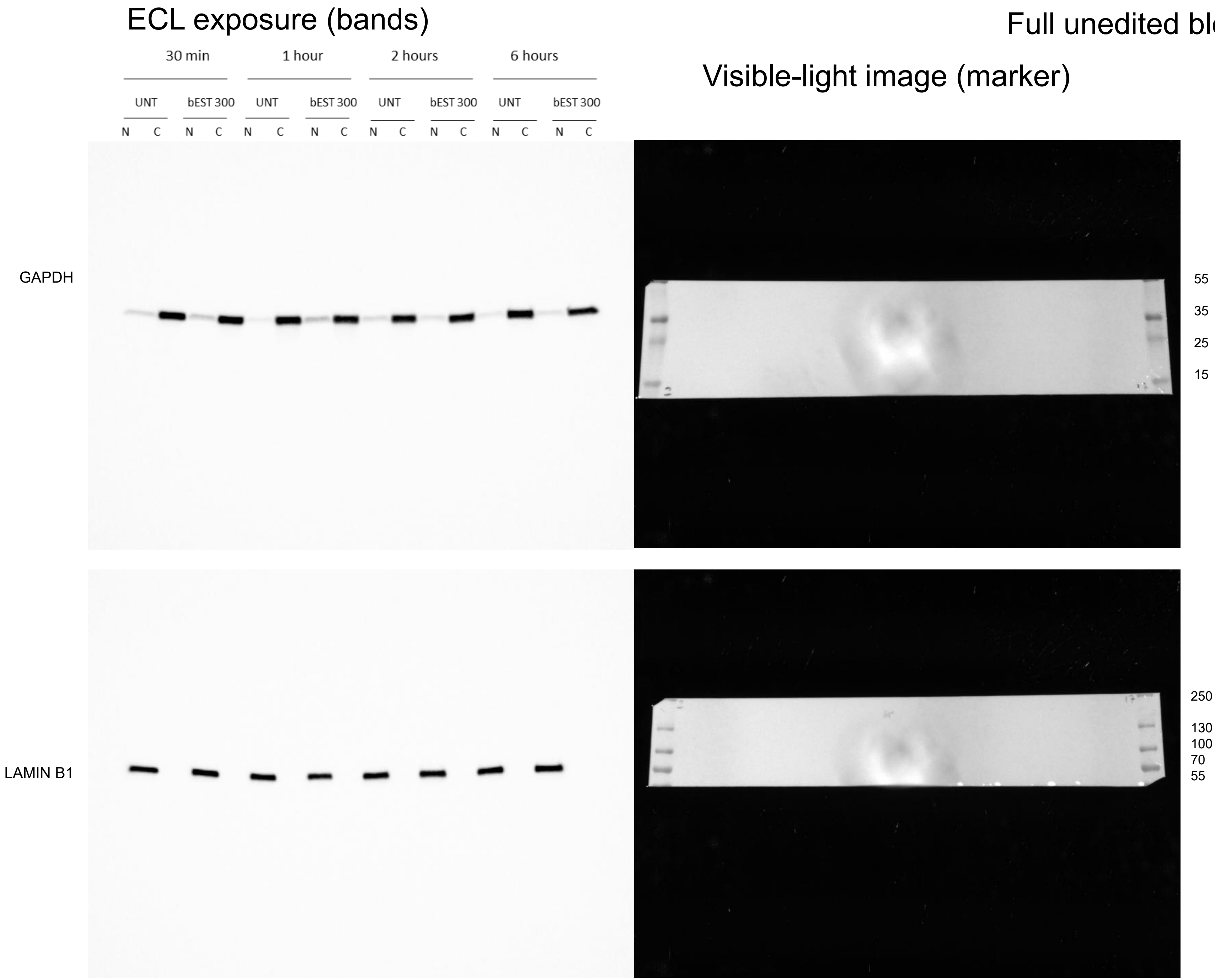

Full unedited blot/gel for Figure S3A

ECL exposure (bands) | Visible-light image (marker)

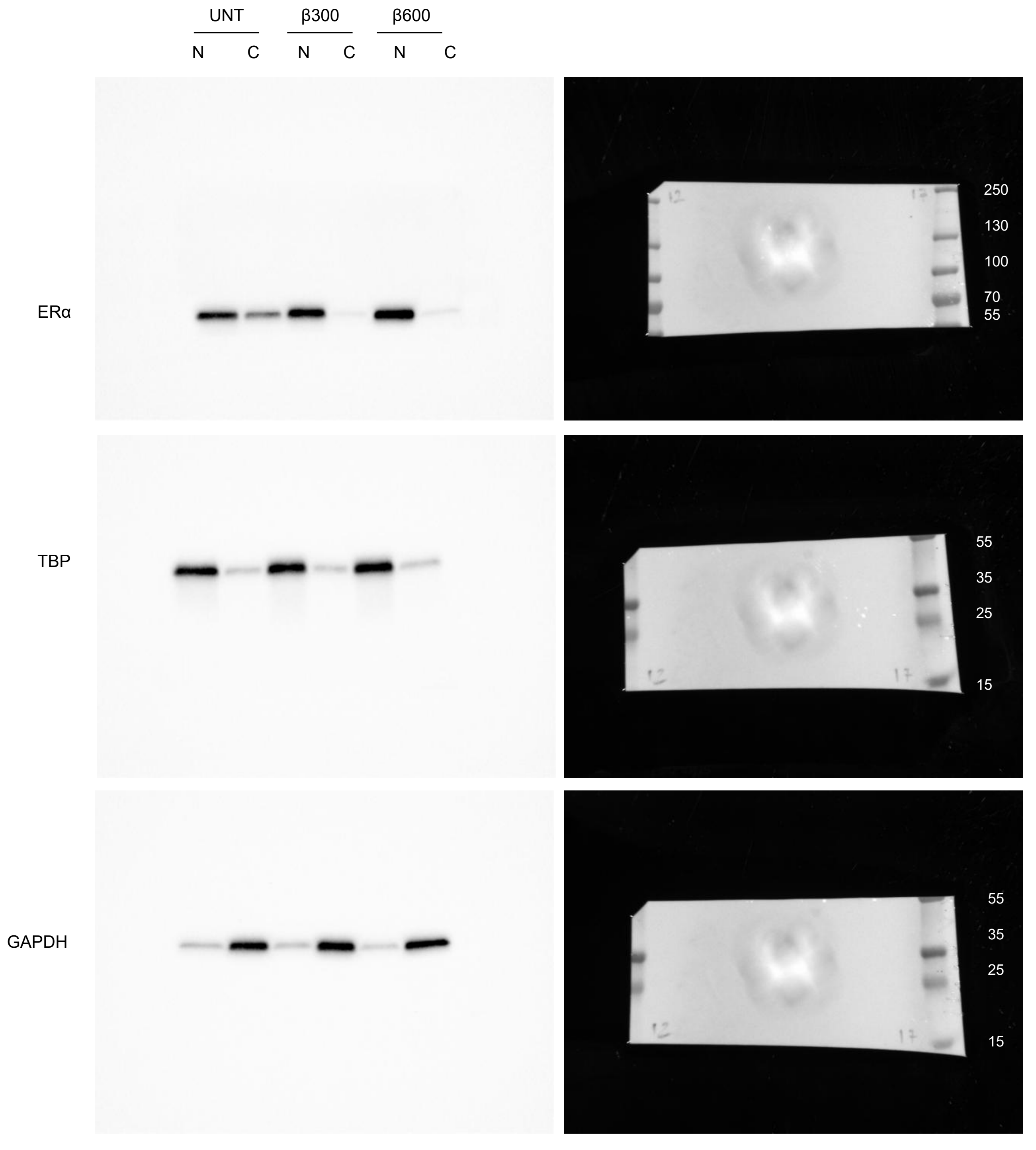

Supplement: Supplementary file 2 [file jciinsight-11-198415-s292.pdf]
